# Small spot size versus large spot size: Effect on plan quality for lung cancer in pencil beam scanning proton therapy

**DOI:** 10.1002/acm2.13512

**Published:** 2022-01-06

**Authors:** Suresh Rana, Anatoly B. Rosenfeld

**Affiliations:** ^1^ Department of Medical Physics The Oklahoma Proton Center Oklahoma City Oklahoma USA; ^2^ Department of Radiation Oncology Boca Raton Regional Hospital Lynn Cancer Institute, Baptist Health South Florida Boca Raton Florida USA; ^3^ Department of Radiation Oncology Herbert Wertheim College of Medicine Florida International University Miami Florida USA; ^4^ Centre for Medical Radiation Physics University of Wollongong Wollongong Australia

**Keywords:** interplay, lung cancer, NTCP, pencil beam scanning, plan robustness, proton therapy

## Abstract

**Purpose:**

The purpose of the current study was to evaluate the impact of spot size on the interplay effect, plan robustness, and dose to the organs at risk for lung cancer plans in pencil beam scanning (PBS) proton therapy

**Methods:**

The current retrospective study included 13 lung cancer patients. For each patient, small spot (∼3 mm) plans and large spot (∼8 mm) plans were generated. The Monte Carlo algorithm was used for both robust plan optimization and final dose calculations. Each plan was normalized, such that 99% of the clinical target volume (CTV) received 99% of the prescription dose. Interplay effect was evaluated for treatment delivery starting in two different breathing phases (T0 and T50). Plan robustness was investigated for 12 perturbed scenarios, which combined the isocenter shift and range uncertainty. The nominal and worst‐case scenario (WCS) results were recorded for each treatment plan. Equivalent uniform dose (EUD) and normal tissue complication probability (NTCP) were evaluated for the total lung, heart, and esophagus.

**Results:**

In comparison to large spot plans, the WCS values of small spot plans at CTV D_95%_, D_96%_, D_97%_, D_98%_, and D_99%_ were higher with the average differences of 2.2% (range, 0.3%–3.7%), 2.3% (range, 0.5%–4.0%), 2.6% (range, 0.6%–4.4%), 2.7% (range, 0.9%–5.2%), and 2.7% (range, 0.3%–6.0%), respectively. The nominal and WCS mean dose and EUD for the esophagus, heart, and total lung were higher in large spot plans. The difference in NTCP between large spot and small spot plans was up to 1.9% for the total lung, up to 0.3% for the heart, and up to 32.8% for the esophagus. For robustness acceptance criteria of CTV D_95%_ ≥ 98% of the prescription dose, seven small spot plans had all 12 perturbed scenarios meeting the criteria, whereas, for 13 large spot plans, there were ≥2 scenarios failing to meet the criteria. Interplay results showed that, on average, the target coverage in large spot plans was higher by 1.5% and 0.4% in non‐volumetric and volumetric repainting plans, respectively.

**Conclusion:**

For robustly optimized PBS lung cancer plans in our study, a small spot machine resulted in a more robust CTV against the setup and range errors when compared to a large spot machine. In the absence of volumetric repainting, large spot PBS lung plans were more robust against the interplay effect. The use of a volumetric repainting technique in both small and large spot PBS lung plans led to comparable interplay target coverage.

## 1 INTRODUCTION

Pencil beam scanning (PBS) proton therapy has shown great potential in reducing the dose to the organs at risk (OARs) and improving dose conformality when compared to passive‐scattering proton therapy.^1^ However, uncertainties such as patient setup, beam range, and the interplay between tumor motion and proton beam delivery can have an impact on PBS treatment plans.^2^ Recently, robust optimization has been implemented within treatment planning systems (TPS) to compensate both setup and range uncertainties.^3^, ^4^ Robustly optimized plans are evaluated in terms of plan robustness by simulating the range uncertainty due to the computed tomography (CT) calibration error and isocenter shift of the patient due to inter‐fraction variations in the patient's position.^5^

The spot size of a pencil proton beam can influence the plan quality and robustness in PBS proton therapy.^6^, ^7^, ^8^, ^9^, ^10^ Plan robustness becomes more critical for the PBS proton lung cancer treatment because the proton beam needs to transverse low‐ and high‐density interfaces in its path, and lung tumor volume may contain large density variations. The evaluation of plan robustness for PBS proton treatment is equally important.^11^ Robust optimization can take into account the combination of setup and range errors and minimize the impact of these errors on the clinical target volume (CTV) coverage and dose to the OARs.^12^, ^13^ The literature addressing the impact of spot size on the plan robustness (setup and range errors) for PBS lung cancer is limited. Liu et al.^9^ investigated the effect of spot size on plan robustness for PBS lung plans and reported that results were similar in a small spot and large spot plans. However, Liu et al.^9^ evaluated the setup and range uncertainties separately, whereas, in a real clinical situation, it is possible that setup errors can occur in conjunction with the range error.

Early investigators have compared small versus large spot sizes to mitigate the interplay effect. Liu et al.^9^ demonstrated that small spot and large spot machines produced comparable interplay effects in 10 lung cancer patients. By contrast, Grassberger et al.^7^ showed that large spot plans are more robust to motion effects due to reduced interplay effect when compared to the small spot plans. In a different study by Grassberger et al.,^14^ it was shown that large spot size plans needed two repaintings (either layer or volumetric) to restore the dose, whereas small spot size plans needed 2 to 6 repaintings to mitigate the interplay effect. Liu et al.^9^ used a synchrotron‐based spot scanning system (Hitachi ProBeat; Hitachi Ltd, Tokyo, Japan), whereas the study by Grassberger et al.^7^ was based on the earlier version of the IBA machine at Massachusetts General Hospital. One of the differences between these two studies was that Grassberger et al.^7^, ^14^ used a non‐robust optimization technique for treatment planning, whereas Liu et al.^9^ generated all plans using a robust optimization technique.

Recently, volumetric repainting technique in an alternating order has been made available on the ProteusPLUS IBA proton machines with a dedicated PBS nozzle.^15^ The use of repainting techniques such as layer and volumetric can reduce the interplay effect by averaging out of hot and cold spots.^16^ The alternating order in volumetric repainting allows beam delivery sequences in “down” and “up” directions – which means the beam can be delivered from the highest energy layer to the lowest energy layer as well as from the lowest energy layer to the highest energy layer. A faster energy layer switching in the “down” and “up” directions is achieved by using a magnetic field regulation feature. In previous studies,^15^, ^17^ experiments were performed investigating the impact of magnetic field regulation in conjunction with the volumetric repainting technique on the spot size, spot position, and range in PBS proton therapy. In a separate publication,^18^ a small spot size beam model was used to investigate the volumetric repainting technique in mitigating the interplay effect for 4D robustly optimized PBS proton lung plans. However, the impact of large spot size on plan robustness and interplay effect was not addressed in that study.^18^

In recent years, new proton therapy centers are equipped with a small spot size. Proton therapy vendors may also provide an additional option for the large spot size on the same machine. This leads to the questions – Is it worth purchasing a PBS proton machine that can provide both small and large spot sizes? How can we utilize small and large spot sizes for the volumetric repainting technique in an alternating order? It is important to understand if there are any dosimetric and radiobiological benefits in using a small spot size versus a large spot size in PBS proton therapy.

The primary aim of the current study was therefore to answer the following questions:

1. What is the impact of spot size on the interplay effect in lung cancer if a proton beam is delivered using a volumetric repainting technique in an alternating order?
2. What is the dosimetric impact of spot size on plan robustness for lung cancer? For plan robustness, the setup error is combined with the range error, whereas the previous study^9^ evaluated the setup and range errors separately.
3. How does the combination of range and setup errors affects the equivalent uniform dose (EUD) and normal tissue complication probability (NTCP) for the OARs in lung cancer?

## 2 MATERIAL AND METHODS

### 2.1 Contouring and treatment planning

In this Institutional Review Board‐approved retrospective dosimetric study, a total of 13 lung cancer patients were selected. Table 1 shows the location of CTV and their motions and volumes in the 13 lung cancer patients. The selection criteria included the presence of four‐dimensional CT (4DCT) scans of ten breathing phases and the lung tumor motion of less than 15 mm. The mediastinum involvement in the target volume was allowed. The 4DCT data set of each patient was anonymized. The tumor motion range ranged from 2.2 to 13.2 mm. The CTV was generated by an isotropic margin of 5 mm around the internal gross tumor volume (IGTV). The IGTV is then overridden with the density of the water.^1^ The average intensity projection CT was used for treatment planning. Treatment plans were generated in RayStation TPS (clinical version 9B; RaySearch Laboratories, Stockholm, Sweden) based on a single field uniform dose (SFUD) technique for a total dose of 70 Gy(RBE) with a fractional dose of 2 Gy(RBE) using an average RBE of 1.1. For a given patient, treatment planning was performed in three steps.

**TABLE 1:** The location of clinical target volume (CTV) and its motion and volume in 13 lung cancer patients

Patient #	CTV (cc)	Motion (mm)	Location	Mediastinum involvement
1	174.6	2.7	RL	Yes
2	103.9	4.7	LUL	No
3	36.5	7.2	RUL	No
4	181.0	13.2	RLL	No
5	24.4	4.8	LUL	No
6	34.2	5.8	LLL	No
7	23.2	4.2	RL	No
8	22.1	8.8	RLL	No
9	26.0	10.1	RLL	No
10	39.3	10.2	RLL	No
11	366.3	2.5	LL	Yes
12	186.8	2.2	RL	Yes
13	26.5	8.1	RLL	No

Abbreviations: LL, left lung; LLL, left lower lobe; LUL, left upper lobe; RL, right lung; RLL, right lower lobe; RUL, right upper lobe.

First, a small spot beam model (1*σ* = 3 mm at isocenter for energy 226.5 MeV) was used to generate small spot plans with no repainting (SSNR). This model is based on the IBA ProteusPLUS proton therapy system with a dedicated PBS nozzle.^19^ For each patient, the optimal beam angles were selected based on the location of the CTV and OARs. The number of beams in a treatment plan varied from 2–3. Treatment plans were robustly optimized (Monte Carlo; 10 000 ions/spot; SFUD technique) on the CTV using 5 mm setup uncertainty and 3.5% range uncertainty^3^ with a goal of 99% of the CTV receiving at least 99% of the prescription dose. Once the final dose calculations (Monte Carlo; 0.5% statistical uncertainty; 3 mm grid size) were completed, SSNR plans were normalized, such that CTV D_99% _= 6930 cGy(RBE).

**FIGURE 1:**
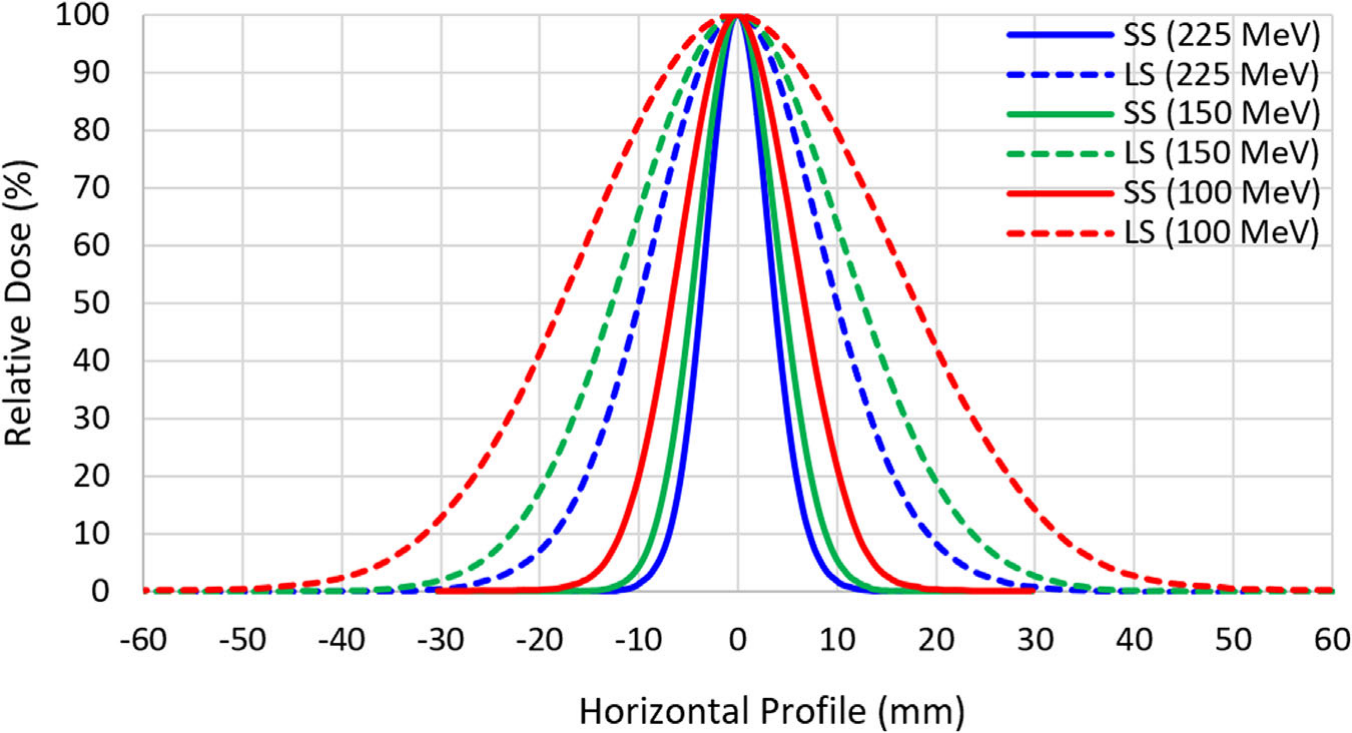
Example of spot profiles from the small spot and large spot models at the isocenter for 100, 150, and 225 MeV

Second, a large spot beam model (1σ = 8 mm at isocenter for energy 226.5 MeV) was used to generate large spot plans with no repainting (LSNR). Spot profiles in a large spot beam model were generated by scaling the spot profiles from a small spot beam model (Figure 1) Other beam model components such as absolute dose output and integrated depth doses (IDDs) remained identical in large spot and small spot beam models. For a given patient, an SSNR plan was copied and switched to a large spot beam model. The plan was then robustly re‐optimized using the same settings like the ones in small spot plan optimization. Also, the number of treatment fields, optimization settings, and optimization objectives remained identical in large spot and small spot plans. To be consistent with SSNR plans, all LSNR plans were calculated with Monte Carlo (0.5% statistical uncertainty; 3 mm grid size) and normalized, such that 99% of the CTV received 6930 cGy(RBE).

**FIGURE 2:**
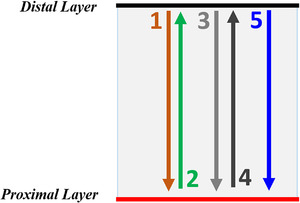
Beam delivery directions in a volumetric repainting plan with an alternating order; Note: beam delivery starts from the distal energy layer to the proximal energy layer, and then follows an alternating order. All plans in the current study included five repaintings

Third, for a given patient, an SSNR was used to generate a volumetric repainting plan with five continuous paintings in an alternating order^15^ (hereafter referred to as SSVR plans). Dose distributions in nominal SSNR and SSVR plans were identical. Figure 2 shows the schematic of the volumetric repainting technique in an alternating order. Similarly, an LSNR plan was used to generate a volumetric repainting plan with five paintings in an alternating order (hereafter referred to as LSVR plans). It was verified there was no change in the dose distributions when generating an LSVR plan from the LSNR plan. In order to ensure the deliverability of spots on the machine, a minimum monitor unit (MU) of 0.015 was applied to all SSVR and LSVR plans. All volumetric repainting plans were generated based on the methodology described by Engwall et al.^12^, ^13^ Prior to dose computation for robustness testing, both sets of plans (SSVR and LSVR) were verified, such that CTV D_99% _= 6930 cGy(RBE).

### 2.2 Robustness analysis

Plan robustness was investigated for 12 perturbed scenarios, which combined the isocenter shift and range uncertainty. The isocenter of the patient was shifted by 5 mm in the superior‐inferior, anterior‐posterior, and right‐left directions, whereas ±3.5% was used for the range uncertainty. The following metrics were used to evaluate the worst‐case scenario (WCS) values based on 12 perturbed scenarios.

1. CTV: D_95%_, D_96%_, D_97%_, D_98%_, and D_99%_.
2. CTV: D_0.03cc_
3. CTV Homogeneity Index (HI): D_99%_/D_1%_
4. Total lung: D_mean_, V_20_, and V_5_ (note: total lung includes both lungs except CTV)
5. Heart: D_mean_
6. Esophagus: D_mean_
7. Spinal Cord: D_0.03cc_

The difference (Δ) between SSVR and LSVR plans was calculated using Equation (1).

(1) $$\Delta \;D_{x}=\left(D_{x}^{\mathrm{LSVR}}-D_{x}^{\mathrm{SSVR}}\right)$$

where, $D_{x}$ = dosimetric metric (e.g., D_mean_, V_20_, etc.); $D_{x}^{\mathrm{LSVR}}$ = dosimetric result of $D_{x}$ in LSVR plan; $D_{x}^{SSVR}$ = dosimetric result of $D_{x}$ in SSVR plan;

### 2.3 Radiobiological analysis

EUD and NTCP evaluation was performed using the dose‐volume histograms (DVHs) of the SSVR and LSVR plans. EUD is based on Niemierko's phenomenological model.^20^, ^21^ To estimate the clinical outcomes of the SSVR and LSVR plans, the EUD‐based NTCP^20^, ^21^ was calculated for all perturbed scenarios. The Total lung, heart, and esophagus were evaluated for the endpoints of pneumonitis, pericarditis, and esophagitis, respectively.^20^, ^21^ The difference (Δ) in radiobiological results was evaluated using Equation (1).

(2) $$\mathrm{EUD}={\left(\sum_{i=1}\left(v_{i}{\mathrm{EQD}}_{i}^{a}\right)\right)}^{\frac{1}{a}}$$

(3) $$\mathrm{EQD}=D\times \left(\frac{\frac{\alpha }{\beta }+\frac{D}{n_{f}}}{\frac{\alpha }{\beta }+2}\right)$$

(4) $$\mathrm{NTCP}=\frac{1}{1+{\left(\frac{TD_{50}}{\mathrm{EUD}}\right)}^{4\gamma_{50}}}$$

where *a *= unit‐less model parameter that is specific to the normal structure or tumor of interest

*v_i_ *= unit‐less *i* ^th^ partial volume receiving dose D*_i_* in Gy

EQD = biologically equivalent physical dose of 2 Gy

*n_f_ *= number of fractions

*d_f_ = D/n_f_ *= dose per fraction size of the treatment course

*TD_50_ *= tolerance dose for a 50% complication rate at a specific time interval when the whole organ of interest is homogeneously irradiated

*γ_50_ *= unit‐less model parameter that is specific to the normal tissue of interest and describes the slope of the dose‐response curve

The values of *a*, *γ_50_*, and *TD_50_* for OARs were obtained from the published literature.^22^, ^23^, ^24^

### 2.4 Interplay effect analysis

The interplay effect was studied in RayStation TPS (clinical version 9B) using the methodology described by Engwall et al.^12^, ^13^ First, deformable registration was performed between the average intensity projection CT and breathing phases from the 4DCT data set using ANAtomically CONstrained Deformation Algorithm.^12^ For the interplay effect analysis, we used the energy layer switching time of 1 s and spot delivery time of 4.0 ms/MU. The motion speed between spots was set to 250 cm/s. The analysis was performed with an assumption of treatment delivery starting in two phases: 0% representing end‐inhalation (*T*_0_) and 50% representing end‐exhalation (*T*_50_). The spots in a treatment plan were distributed over 10 breathing phases according to starting phase (*T*_0_ and *T*_50_) for the beam delivery and spot timings as mentioned above. The dose was computed on ten different breathing phases based on the spot distribution.^12^, ^13^ This was followed by mapping of the dose to the reference phase through deformable registration.^12^, ^13^ The final step included the accumulation of the mapped doses on the reference phase.^12^, ^13^ The interplay DVHs were utilized to evaluate CTV D_95%_, D_99%_, and HI.

## 3 RESULTS

### 3.1 Target volume

#### 3.1.1 Plan robustness

**FIGURE 3:**
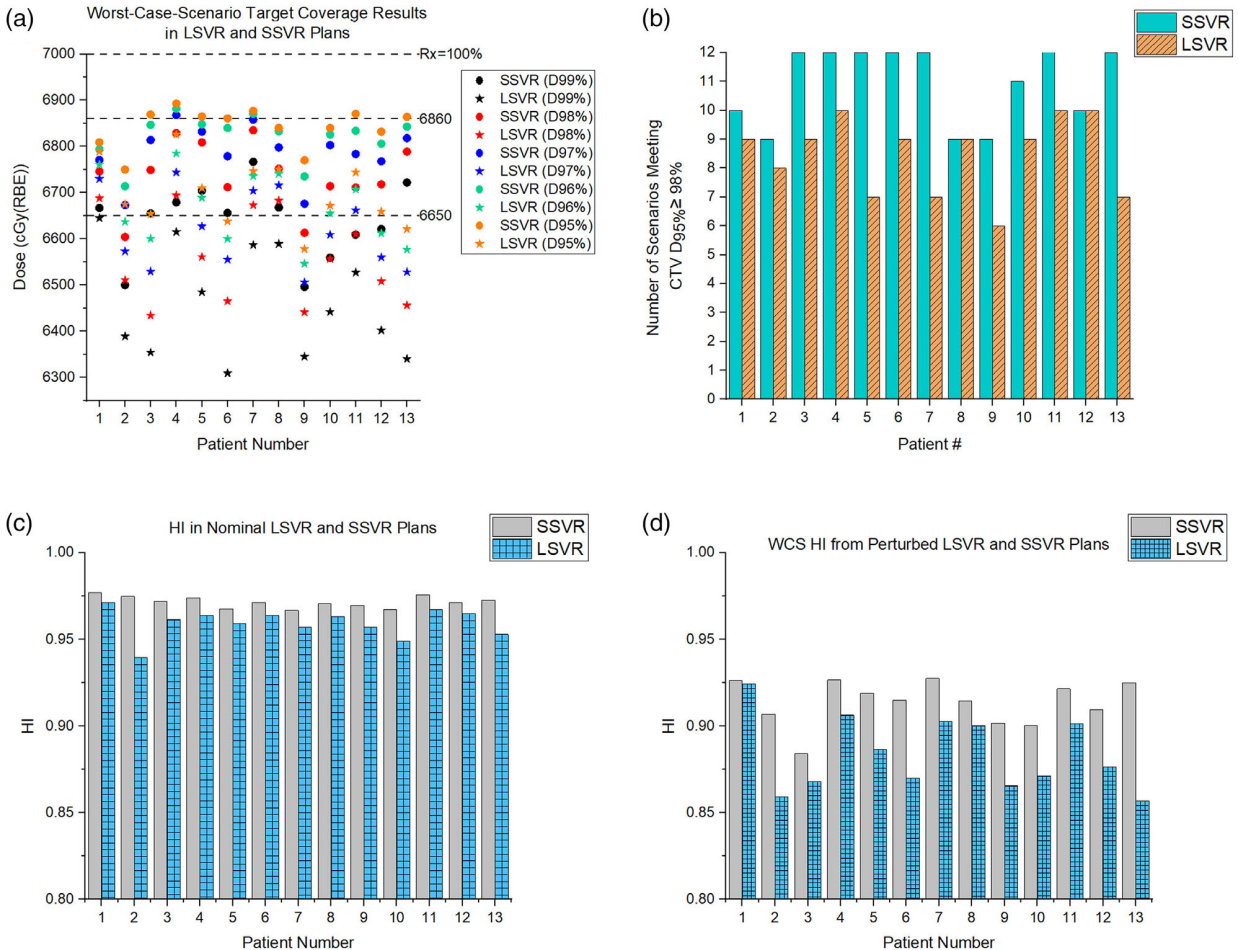
(a) The worst‐case scenario (WCS) values in the large spot plans with five volumetric repaintings (LSVR) and small spot plans with five volumetric repaintings (SSVR) at different dosimetric metrics; (b) Number of perturbed scenarios passing meeting the robustness criteria in LSVR and SSVR plans; (c) Nominal homogeneity index (HI) in LSVR and SSVR plans; and (d) WCS HI in LSVR and SSVR plans

Figure 3a shows the WCS results of the CTV D_95%_, D_96%_, D_97%_, D_98%_, and D_99%_. For a given dosimetric metric, the WCS result in the SSVR plan (denoted by a circle in Figure 3a) was higher than in the LSVR plan (denoted by a star in Figure 3a), thus clearly indicating that SSVR plans were more robust than LSVR plans. Specifically, on average, the WCS values at D_95%_, D_96%_, D_97%_, D_98%_, and D_99%_ were higher by 2.2% (range, 0.3%–3.7%), 2.3% (range, 0.5%–4.0%), 2.6% (range, 0.6%–4.4%), 2.7% (range, 0.9%–5.2%), and 2.7% (range, 0.3%–6.0%), respectively. For robustness acceptance criteria of D_95%_ ≥ 6860 cGy(RBE), seven SSVR plans had all 12 perturbed scenarios meeting the criteria, whereas, for 13 LSVR plans, more than two scenarios were failing to meet the criteria. For treatment plans failing to meet the robustness criteria for all scenarios (Figure 3b), the number of scenarios that met the criteria ranged from 9 to 11 in SSVR plans (*n* = 6) and from 6 to 10 in LSVR plans (*n* = 13). Hot spot evaluation showed that the average difference in WCS values of D_0.03cc_ was lower in SSVR plans by 0.4 ± 1.4%. Figure 3c,d shows the nominal HI and WCS HI of perturbed scenarios, respectively, for all 13 patients. For both nominal and perturbed scenarios, small spot plans produced more homogenous dose distributions compared to large spot plans. Specifically, for a nominal scenario, dose homogeneity was superior in SSVR plans (HI = 0.97) than in LSVR plans (HI = 0.95). A similar trend was observed for the perturbed scenarios. The WCS HI was 0.91 and 0.88 in SSVR and LSVR, respectively.

#### 3.1.2 Interplay effect

**FIGURE 4:**
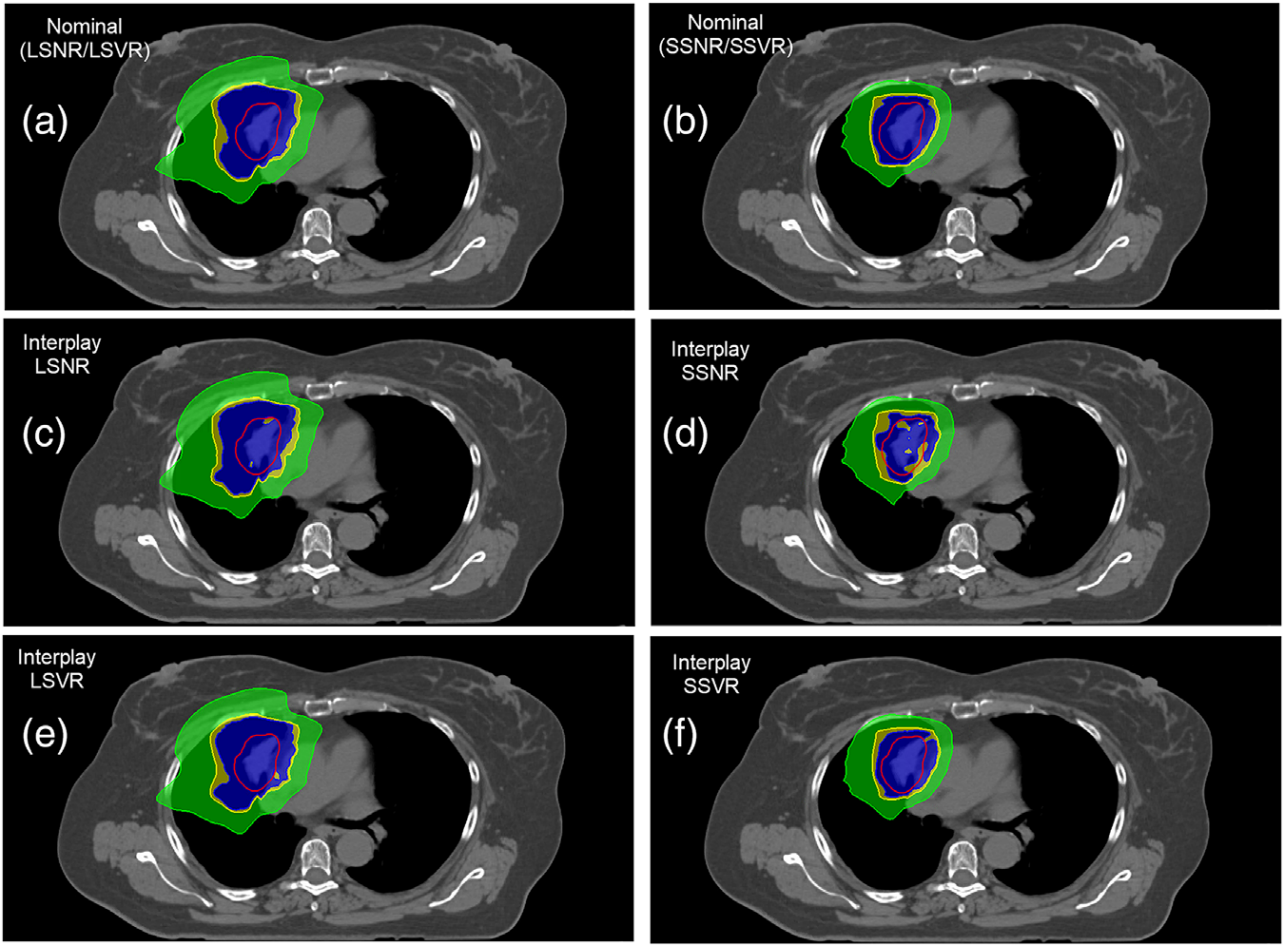
(a,b) Nominal dose distributions in large spot and small spot plans with and without volumetric repainting technique in an example patient; (c,d) Interplay dose distributions without volumetric repainting in large spot and small spot plans in the same patient; (e,f) Interplay dose distributions with volumetric repainting in large spot and small spot plans in the same patient. Note: Red contour = CTV, Blue = 6930 cGy(RBE), yellow = 6650 cGy(RBE); green = 3000 cGy(RBE); LSNR = large spot plan with no volumetric repainting, SSNR = small spot plan with no volumetric repainting, LSVR = large spot plan with five volumetric repaintings, SSVR = small spot plan with five volumetric repaintings

**FIGURE 5:**
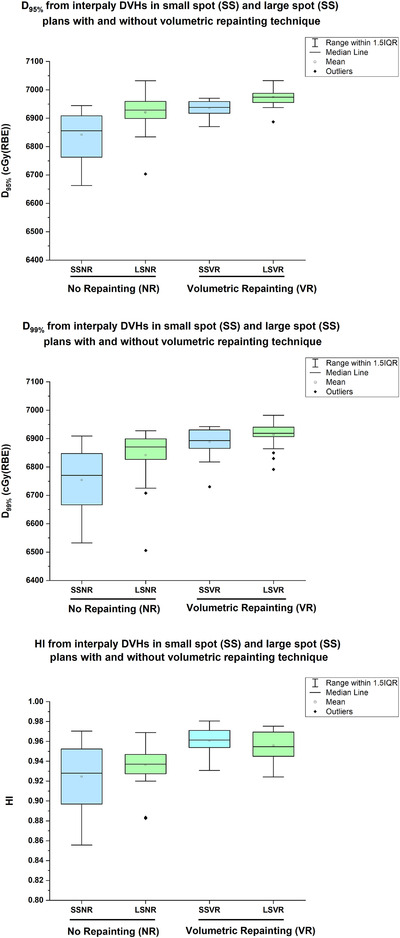
Difference in clinical target volume (CTV) D_95%_, D_99%_, and homogeneity index (HI) from interplay dose‐volume histograms (DVHs) between small spot and large spot plans

Figure 4 illustrates an example of nominal and interplay dose distributions in the small spot and large spot plans with and without volumetric repainting technique. Figure 5 provides the difference in CTV D_95%_ and D_99%_ from interplay DVHs between small spot and large spot plans. In the absence of volumetric repainting, large spot plans were found to be more robust to interplay effect when compared to small spot plans. Specifically, the average differences in D_95%_ and D_99%_ between LSNR and SSNR plans were 1.2 ± 1.0% and 1.3 ± 1.4%, respectively. Figure 4c,d illustrates the interplay of dose distributions in LSNR and SSNR plans, respectively. The loss of CTV D_99%_ (blue color) is clearly visible in interplay SSNR dose distribution as shown in Figure 4d. However, after utilizing the volumetric repainting technique in both sets of plans, the interplay dose distributions between small and large spot plans were found to be comparable. The average differences in D_95%_ and D_99%_ between LSVR and SSVR plans were 0.4 ± 0.6% and 0.4 ± 0.8%, respectively. Figure 4f illustrates that the target coverage loss was recovered in interplay SSVR dose distribution after applying five volumetric repaintings. Figure 5 shows the summary of HI results from interplay DVHs of small spot and large spot plans. If no volumetric repainting technique was used, the HI in LSNR was slightly better than in SSNR plans (0.94 ± 0.02 vs. 0.92 ± 0.03). The use of the volumetric repainting technique resulted in similar HI results in LSVR (0.95 ± 0.01) and SSVR (0.96 ± 0.01) plans.

### 3.2 Organs at risk

**FIGURE 6:**
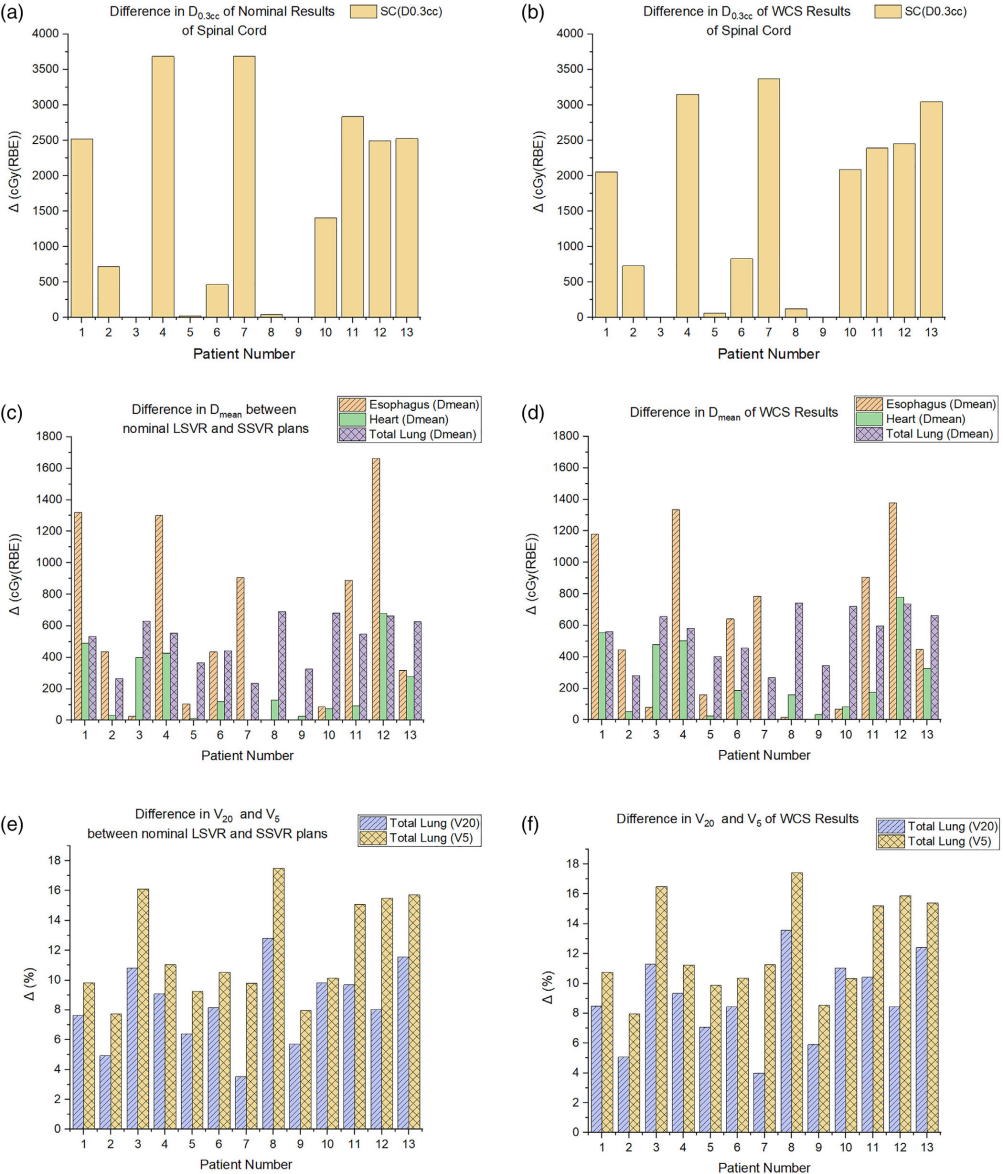
(a) Difference in D_0.03cc_ of nominal results for the spinal cord between large spot plans with five volumetric repaintings (LSVR) and small spot plans with five volumetric repaintings (SSVR); (b) Difference in D_0.03cc_ of worst‐case scenario (WCS) results for the spinal cord between LSVR and SSVR plans; (c) Difference in D_mean_ of nominal results for the organs at risk (OARs; esophagus, heart, and total lung) between LSVR and SSVR plans; (d) Difference in D_mean_ of WCS results for the OARs (esophagus, heart, and total lung) between LSVR and SSVR plans; (e) Difference in V_20_ and V_5_ of nominal results for the total lung between LSVR ad SSVR plans; (f) Difference in V_20_ and V_5_ of WCS results for the total lung between LSVR ad SSVR plans

Figure 6a shows the difference in D_0.03cc_ to the spinal cord in nominal plans. The difference in WCS D_0.03cc_ to the spinal cord is presented in Figure 6b. For a nominal scenario, a large spot beam model resulted in a higher D_0.03cc_ by an average difference of 1570 cGy(RBE). A similar observation was made for the WCS D_0.03cc_ to the spinal cord (Δ = 1562 cGy[RBE]). Both the nominal and WCS D_mean_ to the esophagus, heart, and total lung was higher in LSVR plans. (Figures 6c,d) Specifically, for a nominal scenario, the average difference for the esophagus, heart, and total lung was 576 cGy(RBE), 212 cGy(RBE), and 505 cGy(RBE), respectively, whereas the difference in WCS values from the perturbed scenarios was 572 cGy(RBE), 258 cGy(RBE), and 549 cGy(RBE), respectively. The average difference in V_20_ and V_5_ of the total lung for a nominal scenario was 8.3% and 12.0%, respectively. (Figure 6e) A similar difference was found for the WCS V_20_ (Δ = 8.9%) and V_5_ (12.4%) of the total lung. (Figure 6f)

**FIGURE 7:**
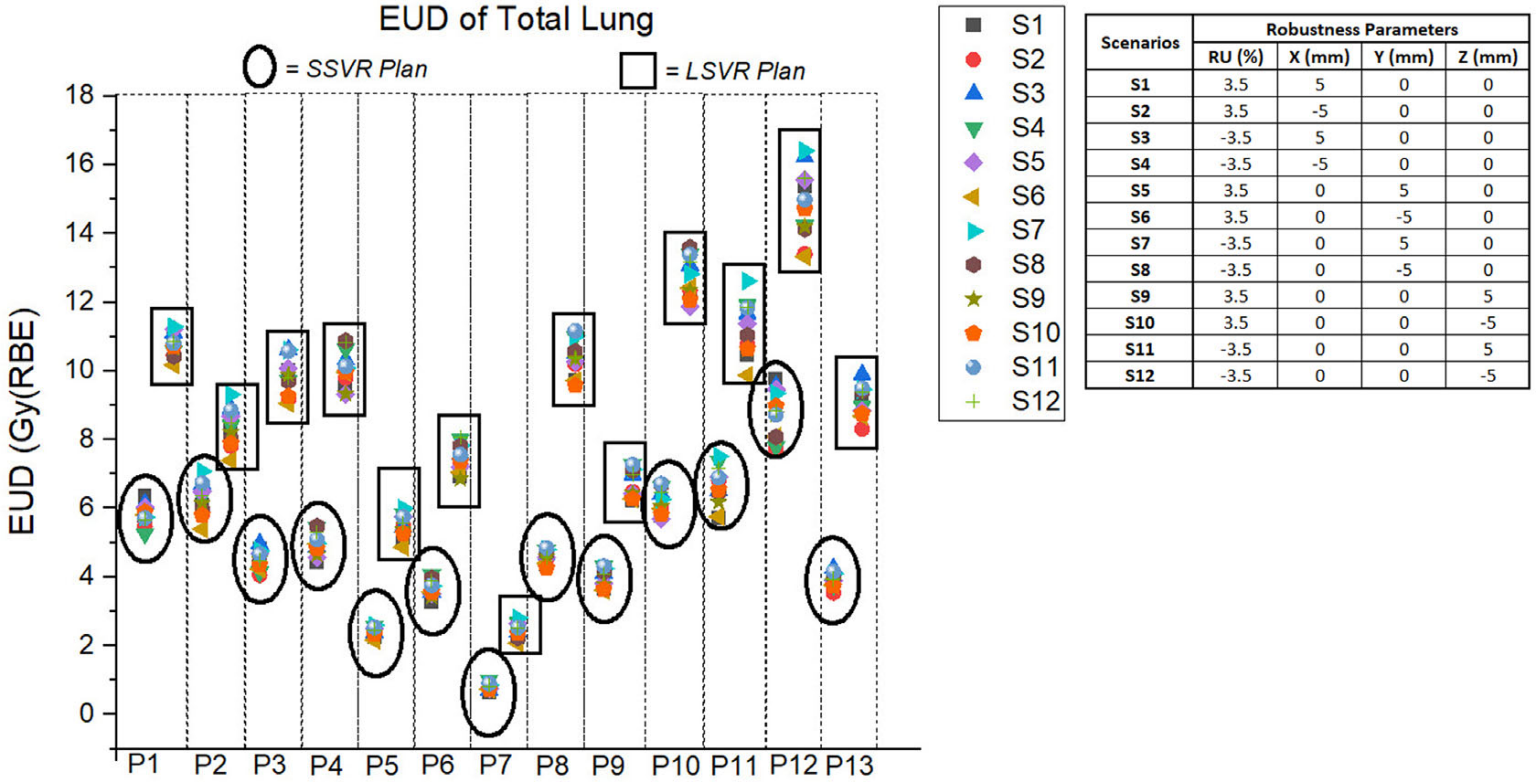
Equivalent uniform dose (EUD) of the total lung for 12 perturbed scenarios (S1–S12) in large spot plans with five volumetric repaintings (LSVR) and small spot plans with five volumetric repaintings (SSVR) of all 13 patients

Table 2 provides the WCS results of EUD and NTCP for the total lung, heart, and esophagus. Figure 7 shows the total lung EUD for 12 perturbed scenarios in all 13 patients. On average, the difference in EUD was 469 cGy(RBE) for the total lung, 495 cGy(RBE) for the heart, and 557 cGy(RBE) for the esophagus. For the NTCP, the difference ranged from 0% to 1.91% for the total lung, from 0% to 0.34% for the heart, and from 0% to 32.80% for the esophagus.

**TABLE 2:** Equivalent uniform dose (EUD) and normal tissue complication probability (NTCP) for the total lung, heart, and esophagus in spot plan with five volumetric repaintings (LSVR) and small spot plan with five volumetric repaintings (SSVR)

Total lung						
	EUD (cGy(RBE))			NTCP (%)		
Patient #	SSVR	LSVR	Δ	SSVR	LSVR	Δ
1	636	1128	492	0.00	0.10	0.10
2	708	931	223	0.00	0.02	0.02
3	497	1064	566	0.00	0.06	0.06
4	548	1087	540	0.00	0.07	0.07
5	259	600	341	0.00	0.00	0.00
6	407	806	400	0.00	0.01	0.01
7	99	281	181	0.00	0.00	0.00
8	484	1117	633	0.00	0.09	0.09
9	431	728	296	0.00	0.00	0.00
10	671	1359	688	0.00	0.44	0.43
11	752	1262	510	0.00	0.24	0.24
12	977	1641	664	0.03	1.94	1.91
13	426	990	564	0.00	0.03	0.03

Heart						
	EUD (cGy(RBE))			NTCP (%)		
Patient #	SSVR	LSVR	Δ	SSVR	LSVR	Δ
1	2200	2866	666	0.01	0.20	0.20
2	49	331	282	0.00	0.00	0.00
3	1831	2644	813	0.00	0.08	0.08
4	1953	2705	752	0.00	0.10	0.10
5	4	229	225	0.00	0.00	0.00
6	658	1508	850	0.00	0.00	0.00
7	0	7	7	0.00	0.00	0.00
8	1097	1441	344	0.00	0.00	0.00
9	250	414	164	0.00	0.00	0.00
10	681	1041	361	0.00	0.00	0.00
11	32	631	599	0.00	0.00	0.00
12	2356	3003	647	0.02	0.36	0.34
13	1655	2386	731	0.00	0.02	0.02

Esophagus						
	EUD (cGy(RBE))			NTCP (%)		
Patient #	SSVR	LSVR	Δ	SSVR	LSVR	Δ
1	4002	4896	894	12.57	41.91	29.34
2	981	1520	538	0.00	0.01	0.01
3	3	137	133	0.00	0.00	0.00
4	1779	3266	1487	0.02	2.75	2.73
5	387	629	242	0.00	0.00	0.00
6	463	1503	1040	0.00	0.01	0.01
7	3184	3722	538	2.25	7.44	5.19
8	0	34	34	0.00	0.00	0.00
9	0	0	0	0.00	0.00	0.00
10	535	597	63	0.00	0.00	0.00
11	4399	4870	471	23.45	40.87	17.42
12	3529	4792	1263	5.00	37.79	32.80
13	198	738	540	0.00	0.00	0.00

## 4 DISCUSSION

The current study was performed to determine if there is a dosimetric and radiobiological benefit of using small spot size versus large spot size for lung plans in PBS proton therapy. All treatment plans in our study were robustly optimized against the setup and range uncertainties. Also, we assumed that range uncertainties occurred in conjunction with an isocentric shift of the patient in a given direction (superior‐inferior, anterior‐posterior, and right‐left), thus resulting in 12 perturbed scenarios. In order to make a fair plan comparison, both sets of plans (SSVR and LSVR) were normalized, such that CTV D_99% _= 6930 cGy(RBE). The WCS results at various dosimetric metrics of the CTV showed that the target coverage in LSVR plans was reduced by up to 6%. In comparison to LSVR plans, the number of scenarios passing the robustness criteria (D_95%_ ≥ 6860 cGy[RBE]) in each patient was higher in SSVR plans, except for patients #8 and #12 – both plans (SSVR and LSVR) produced the same number of scenarios passing the criteria for these two patients.

Recently, new proton centers are installing machines with smaller spot sizes. Plan robustness results from our study demonstrated that if a lung plan is robustly optimized with a small spot beam model (∼3 mm) and volumetric repainting is applied, it is feasible to generate a treatment plan that is robust against the setup and range errors. However, if an SSVR plan requires the use of a range shifter in the beam path, this will create an air gap between the downstream of a range shifter and patient body/immobilization devices. Since in‐air spot size increases with an increase in the air gap,^25^, ^26^, ^27^ it is recommended to minimize the air gap between the range shifter and patient. This will allow maintaining the robustness of the target volume against the setup and range errors as well as decrease the EUD for the OARs.

The OARs results from our study showed that the smaller spot model produced a lower dose to the normal total lung, heart, esophagus, and spinal cord. These findings are in agreement with Liu et al.^9^ We also noticed that the small spot model resulted in a decrease in EUD for the total lung, heart, and esophagus. The NTCPs for the total lung and heart were found to be comparable in both sets of plans, whereas the difference in NTCP of esophagus between large spot and small spot plans was found to vary from 0% to 32.8%. Such a large difference in NTCP of the esophagus between two sets of plans can be attributed to the location of tumor volume. In patients #1 (ΔNTCP = 29.3%), #11 (ΔNTCP = 17.4%), and #12 (ΔNTCP = 32.8%), the mediastinum is included in the CTV. The location of the CTV in the proximity of the esophagus and wider penumbrae from large spots were found to be contributing factors for increased EUD and NTCP of the esophagus in large spot plans of patients #1, #11, and #12. The NTCP results presented in our study include the uncertainty in the calculations. The NTCP calculations included the parameters that are derived from the photon therapy. Proton‐specific radiobiological parameters for lung cancer are needed to obtain more accurate NTCP calculations, which can be correlated to the tissue toxicities.

Although our study was undertaken on the lung disease site, it is relevant to mention plan robustness studies conducted on other disease sites. For instance, Moteabbed et al.^10^ investigated the impact of spot size on 14 patients (seven central nervous system [CNS], four head and neck, two pelvic, and one thoracic solid tumors) and concluded that plan quality improved as the spot size decreased. More recently, Kraan et al.^8^ investigated the impact of spot size on plan robustness in seven patients of different cancer sites (pelvis, chest wall, rectum, chordoma, cardiac, retroperitoneal, and sarcoma). The results from Kraan et al.^8^ showed that small spot plans are more robust against spot size changes than large spot plans.

Treatment of lung cancer using PBS protons can raise the concern of the interplay effect between tumor motion and delivery of pencil proton beams. The interplay effect evaluation from our study provided two major observations. First, if PBS lung plans with motion did not include the volumetric repainting, small spot plans were found to be more sensitive to the interplay compared to large spot plans. A similar observation was reported by Grassberger et al.^7^ in their PBS lung cancer study (no repainting strategies utilized). The repainting techniques were applied by Grassberger et al.^14^ in a separate study with focus on the layer and volumetric repainting techniques. It was demonstrated that the number of repaintings needed in large spot plans was lower than in small spot plans. The robust optimization technique was not applied in their study.^14^ Our second observation was that, after applying five volumetric repaintings, the interplay effect in SSVR and LSVR plans was found to be comparable. Liu et al.^9^ also reported the comparable interplay effect results for small spot and large spot plans using layer repainting strategies. In the current study, we applied a total of five volumetric repaintings across all patients. For a given treatment field in a patient, the beam‐on time and number of energy layers in a small spot versus a large spot plan were found to be similar. Overall, for five volumetric repaintings, beam‐on time per treatment field ranged from 72 to 216 s, whereas the energy layers ranged from 65 to 165. The number of volumetric repaintings needed to reduce the interplay effect could vary from one patient to another.^13^, ^18^ The readers must not assume that five repaintings used in the current study are an ideal number for the volumetric repainting technique in PBS lung cancer plans. Instead, patient‐specific interplay effect evaluation is recommended to determine the optimal number of repaintings.^13^, ^18^

## 5 CONCLUSION

For robustly optimized PBS lung cancer plans in our study, a small spot machine resulted in a more robust CTV against the setup and range errors when compared to a large spot machine. Overall, small spot plans produced lower EUD for the OARs. In the absence of volumetric repainting technique, large spot PBS lung plans were more robust against the interplay effect. However, the use of a volumetric repainting technique in an alternating order in both small and large spot PBS lung cancer plans led to comparable interplay target coverage.

## FUNDING INFORMATION

This study does not include any funding.

## CONFLICT OF INTEREST

The authors declare that they have no conflict of interest.
